# Correlates associated with participation in physical activity among adults: a systematic review of reviews and update

**DOI:** 10.1186/s12889-017-4255-2

**Published:** 2017-04-24

**Authors:** Jaesung Choi, Miyoung Lee, Jong-koo Lee, Daehee Kang, Ji-Yeob Choi

**Affiliations:** 1 Department of Biomedical Sciences, Seoul National University Graduate School, 103 Daehak-ro, Jongno-gu, Seoul, 03080 Korea; 2 College of Physical Education and Sport Science, Kookmin University, 77 Jeongneung-ro, Seongbuk-gu, Seoul, 02707 Korea; 3 JW Lee Center for Global Medicine, Seoul National University College of Medicine, 71 Ihwhajang-gil, Jongno-gu, Seoul, 03087 Korea; 4 Department of Family Medicine, Seoul National University College of Medicine, 101 Daehak-ro, Jongno-gu, Seoul, 03080 Korea; 5 Institute of Environmental Medicine, Seoul National University Medical Research Center, 103 Daehak-ro, Jongno-gu, Seoul, 03080 Korea; 6 Department of Preventive Medicine, Seoul National University College of Medicine, 103 Daehak-ro, Jongno-gu, Seoul, 03080 Korea; 7 Cancer Research Institute, Seoul National University, 103 Daehak-ro, Jongno-gu, Seoul, 03080 Korea

**Keywords:** Physical activity, Epidemiologic factors, Review of reviews

## Abstract

**Background:**

Understanding which factors influence participation in physical activity is important to improve the public health. The aim of the present review of reviews was to summarize and present updated evidence on personal and environmental factors associated with physical activity.

**Methods:**

MEDLINE and EMBASE were searched for reviews published up to 31 Jan*.* 2017 reporting on potential factors of physical activity in adults aged over 18 years. The quality of each review was appraised with the Assessing the Methodological Quality of Systematic Reviews (AMSTAR) checklist. The corrected covered area (CCA) was calculated as a measure of overlap for the primary publications in each review.

**Results:**

Twenty-five articles met the inclusion criteria which reviewed 90 personal and 27 environmental factors. The average quality of the studies was moderate, and the CCA ranged from 0 to 4.3%. For personal factors, self-efficacy was shown as the strongest factor for participation in physical activity (7 out of 9). Intention to exercise, outcome expectation, perceived behavioral control and perceived fitness were positively associated with physical activity in more than 3 reviews, while age and bad status of health or fitness were negatively associated with participation in physical activity in more than 3 reviews. For environmental factors, accessibility to facilities, presence of sidewalks, and aesthetics were positively associated with participation in physical activity.

**Conclusions:**

The findings of this review of reviews suggest that some personal and environmental factors were related with participation in physical activity. However, an association of various factors with physical activity could not be established because of the lack of primary studies to build up the organized evidence. More studies with a prospective design should be conducted to understand the potential causes for physical activity.

**Electronic supplementary material:**

The online version of this article (doi:10.1186/s12889-017-4255-2) contains supplementary material, which is available to authorized users.

## Background

Participation in regular physical activity contributes to health promotion, improving physical fitness, and prevention of non-communicable diseases [1–4]. The international health guideline for physical activity recommends that adults should be doing at least 150 min of moderate-intensity physical activity throughout the week or doing at least 75 min of vigorous-intensity physical activity regardless of the domains of physical activity such as leisure, transportation, occupational, and household chores [5]. However, the level of inactivity is reported to be high globally [6, 7]. Thus, motivating the public to participate in physical activity by finding which factors influence participation in physical activity is important to improve the public health and to mitigate the global burden of chronic diseases.

There are several theories that describe behavioral models of physical activity, and it is common to incorporate ideas from these theories into ecological models. According to an ecological model, factors which influence health behavior consisted of intra-personal, inter-personal, and environmental factors as well as policy [8]. Personal factors include demographic and biological factors, psychological, cognitive and emotional factors, behavioral factors, and social and cultural factors [9]. Environment factors include the facility, neighborhood, safety, home environment, location of region, and climate [10].

Although there has been one meta-analysis of associations between environmental factors and physical activity [11], most factors related to physical activity have been summarized by systematic reviews rather than by meta-analysis because of an insufficient number of primary studies on each factor and distinct analytical methods. In a study by Bauman, the authors conducted a review of reviews which is a capable method of summarizing previous evidence from systematic reviews, with or without synthesis [12, 13]. They reviewed variables as determinants of physical activity in children or adolescent among adults to investigate those factors throughout their life span; however, the variables studied in adults, but not in children or adolescents, were not reviewed [14].

The primary purpose of this study was to summarize and present updated evidence for personal and environmental factors potentially associated with participation in physical activity overall or by the domains of physical activity.

## Methods

### Search strategy and eligibility criteria

**Fig. 1:**
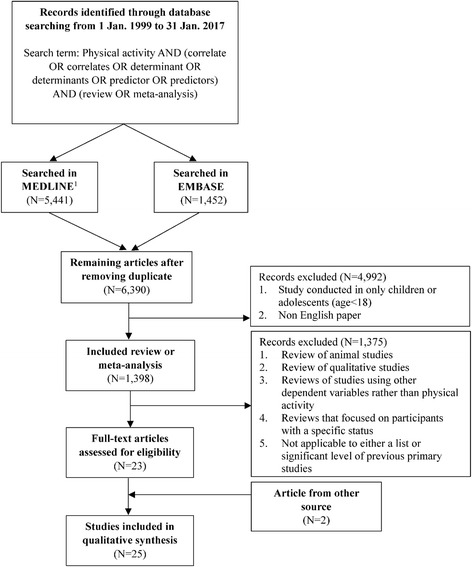
Flow chart of inclusion and exclusion criteria for previous reviews. ^1^Medical subject headings (MeSH) such as ‘Motor Activity’ and ‘Epidemiologic Factors’ were also used in the search strategy

To identify systematic reviews, MEDLINE and EMBASE were searched for quantitative, peer-reviewed studies published up to 31 Jan. 2017 reporting on potential correlates, predictors or determinants of any type of physical activity in adults aged over 18 years (Fig. 1). Search terms indicative of physical activity were used in combination with correlates or determinants. For the adaption of search strategies, specific filters were used from the databases including study design, publication year, language, and age. In MEDLINE, medical subject headings (MeSH) such as ‘motor activity’ and ‘epidemiologic factors’ were also used in the search strategy.

After the removal of reviews that were duplicates in both literature databases or published in a non-English language or that targeted adolescents, the additional following reviews were excluded: 1) reviews of animal studies, 2) reviews of qualitative studies, 3) reviews of studies using other dependent variables rather than physical activity, 4) reviews that focused on participants with a specific status such as cancer, pregnancy, and alcohol use disorder, and 5) studies which did not provide either a list or significant level of previous primary studies because that information was used in the classification of the variables. Reference lists of the included reviews and primary studies in each review were checked to identify any unrevealed studies.

### Rating the methodological quality

To assess the quality of each included review, the 11-item Assessment of Multiple Systematic Review (AMSTAR) checklist was used for the assessment [15]. The measure satisfies inter-observer agreement, reliability, construct validity and feasibility. The quality score ranges from 0 (lowest quality) to 11 (highest quality). In the current study, a review with a 0–2 AMSTAR score was considered as having a low quality, 3–6 as having a moderate quality and 7–11 as having a high quality. The checklist of the AMSTAR score is provided in Additional file 1: Table S1.

### Data extraction

The following characteristics were extracted from the included reviews: report type (e.g., systematic review or meta-analysis), publication year, age of population, number of quantitative studies, outcomes, and proportion of longitudinal studies, and measurement method of physical activity and environmental factors. The domains of physical activity were collected as the outcome if the results of the primary studies included in each review were identifiable by the domains of physical activity.

### Classification of variables

Variables from each review were classified according to the number of primary studies supporting the association or no association and the percentage of expected association among the total number of primary studies (Additional file 1: Table S2) [14]: not a correlate (NC) or not a determinant (ND), inconclusive (IC), a correlate (Cor) or determinant (Det). When more than 50% of the primary studies supporting an association or no association were derived from a longitudinal design, the variables were coded as a determinant rather than a correlate. If the factors were classified as a ‘correlate’ or a ‘determinant’, it was regarded as a definitely associated factor (DAF).

### Corrected Covered Area (CCA)

Because some primary studies were included in more than one review, the summarized results from each review can be biased by those overlaps. To assess this bias, the degree of overlap between reviews was calculated with the Corrected Covered Area (CCA) method. The details of the CCA calculation have been described elsewhere [16]. Briefly, the CCA was calculated with the following equation showing how the primary studies in each review are duplicated:

$$ \mathrm{Corrected}\ \mathrm{Covered}\ \mathrm{Area}\kern0.5em \left(\mathrm{CCA}\right)=\frac{N- r}{rc- r} $$

where N is the sum of the number of primary studies in each review, r is the total number of primary studies, and c is the number of reviews. This measure has been validated in which the number of overlapped primary publications has a strong correlation with the CCA. A CCA score of less than 5% is regarded as a slight overlap, 5–9.9% as moderate overlap, 10–14.9% as high overlap and over 15% as a very high level of overlap [16]. The CCA was estimated for overall personal and environmental factors as well as for the factors classified as DAFs in more than 3 reviews. A study by Duncan et al. [11] was excluded in the CCA calculation because the list of included primary studies was not available.

## Results

A total of 25 reviews with 980 primary studies met the inclusion criteria [9–11, 17–38]. Among those reviews, there were 13 reviews with personal factors [9, 10, 17, 19, 22, 23, 26, 28, 32–34, 36, 38] and 19 reviews with environmental factors [9–11, 18–21, 24, 25, 27–31, 33, 35–38], respectively (Table 1). The number of primary studies for personal factors included in each review ranged from 11 to 91, and the number of primary studies for environmental factors ranged from 3 to 70. Four reviews included only primary studies conducted with a longitudinal design [28, 33, 36, 38]. Thus, the results derived from those reviews were regarded as a determinant rather than as a correlate. Reviews published before 1999 were not considered in the present study because a study by Trost et al. [9] had included and updated the results of those reviews [39–42].

**Table 1:** Characteristics of previous reviews of personal and environmental factors on participation in physical activity

Author	Year	Population age	Publication period of studies	No. of quantitative studies	Outcomes	Proportion of longitudinal studies	No. of assessed factors	Ref
Reviews of personal factors								
Rhodes	1999	≥65	~1999	41	Leisure	14 (34%)	23	[17]
Eyler	2002	≥18 women	1980–2000	81	Overall, leisure, household, transport	0	32	[10]
Trost	2002	≥18	1998–2000	36	Overall	7 (18%)	48	[9]
Plonczynski	2003	≥65 women	1994–2001	15	Overall	1 (6%)	16	[19]
Rhodes	2006	≥18	1969–2006	33	Overall	16 (50%)	6	[23]
Kaewthummanukul	2006	NA	1990–2002	11	Overall	0	22	[22]
Allender	2008	NA	1977–2007	19	Overall	9 (47%)	5	[26]
VanStralen	2009	≥40	1900–2008	54	Overall	54 (100%)	36	[28]
Kirk	2011	18–64	1974–2010	62	Leisure	11 (18%)	6	[32]
Koeneman	2011	≥55	1990–2010	34	Overall, leisure	34 (100%)	31	[33]
Engberg	2012	17–70	1992–2012	34	Leisure	27 (79%)	5	[34]
Rhodes	2015	18–64	2012–2014	78	Overall	75 (100%)	20	[36]
Prince	2016	18–65 women	~2014	91	Overall	91 (100%)	29	[38]
Reviews of environmental factors								
Eyler	2002	≥18	1980–2000	15	Overall, leisure, household	0	13	[10]
Trost	2002	≥18	1998–2000	8	Overall	0	14	[9]
Humpel	2002	Adults	NR	19	Overall	1 (5%)	14	[18]
Plonczynski	2003	≥65 women	1994–2001	5	Overall	0	6	[19]
Cunningham	2004	Adults	1966–2002	27	Overall, leisure, walking,	0	13	[20]
Owen	2004	Adults	~2004	18	Walking	2 (11%)	14	[21]
Duncan^a^	2005	NA	1989-2005	16	Overall	0 (0%)	6	[11]
Tucker	2007	NA	1980–2006	6	Overall	0	2	[24]
Wendel-Vos	2007	≥18	1980–2004	47	Overall	3 (6%)	20	[25]
Saelens	2008	Adults	2005–2006	29	Walking	0	8	[27]
Van Stralen	2009	≥40	1900–2008	13	Overall	13 (100%)	12	[28]
Panter	2010	18–65	1990–2009	43	Transport	0	4	[29]
Koeneman	2011	≥55	1990–2010	3	Leisure, overall	3 (100%)	4	[33]
McCormack	2011	≥18	1996–2010	31	Overall, leisure, walking/cycling, transport	2 (6%)	10	[30]
Van Cauwenberg	2011	Mean > 65	2000–2010	31	Overall, leisure, walking/cycling, transport	3 (10%)	10	[31]
Van Holle	2012	18–65	2000–2011	70	Overall, leisure, walking/cycling, transport	1 (0%)	11	[35]
Rhodes	2015	18–64	2012–2014	12	Overall	12 (100%)	2	[36]
Day	2016	NA	~2014	42	Overall, leisure, transport, occupation	0	12	[37]
Prince	2016	18–65 women	~2014	9	Overall	9 (100%)	6	[38]

^a^Meta-analysis

The quality assessment scores are presented in Additional file 1: Table S1. The AMSTAR score for each review ranged from 2 to 8. Most of the reviews (21 out of 25) were rated as having a moderate quality. Information on study design (checklist 1), literature search strategy (checklist 3) and list of included studies (checklist 5) were provided in most studies. However, information on the status of the publication as an inclusion criterion (checklist 4), the combining methods (checklist 9), publication bias assessment (checklist 10) and conflict of interest of the included studies (checklist 11) were rarely provided.

### Correlates of physical activity overall

A total of 117 factors were reported in the previous reviews. The definitions of each factor are shown in Additional file 1: Table S3 in alphabetical order.

Table 2 lists the relationships between personal factors and physical activity overall. There were 90 personal factors consisting of 24 demographic/biological factors, 40 psychological factors, 13 behavioral factors, and 13 social factors. Among the 90 personal factors, 53 factors were considered as DAFs in more than one of the reviews. For demographic and biological factors, age, gender, ethnicity, marital status, education, income, and employment were assessed in more than half of the reviews (7 out of 13). Among those, age was regarded as a negative DAF in 3 reviews. Bad health or fitness status was assessed in 5 reviews and classified as a negative DAF in 3 reviews. For psychological factors, cognitive and emotional factors, attitude, intention to exercise, outcome expectations, self-efficacy, and stress were assessed in more than half of the reviews. Intention to exercise, outcome expectations, perceived behavioral control, self-efficacy and perceived good fitness were assessed as positive DAFs in more than 3 reviews. Self-efficacy classified as a DAF in 7 reviews had the strongest association with participation in physical activity in this review of reviews. For behavioral factors, smoking was assessed in 7 reviews, which was not determined as a DAF in any of the reviews. For social and cultural factors, there were no variables evaluated in more than half of the reviews.

**Table 2:** Relationships between personal factors and physical activity

	Rhodes (1999) [17]	Eyler (2002) [10]	Trost (2002)^a^ [9]	Plonczynski (2003) [19]	Rhodes (2006) [23]	Kaewthummanukul (2006) [22]	Allender (2008) [26]	Van Stralen (2009) [28]	Kirk (2011) [32]	Koeneman (2011) [33]	Engberg (2012) [34]	Rhodes (2015) [36]	Prince (2016) [38]	No. of DAF/total No.^b^
Demographic and biological factors														
Age	Cor (−)	IC	Cor (−)	IC	-	Cor (−)	-	ND	-	ND	-	ND	-	3/8
Gender, men	Cor (+)	-	Cor (+)	-	-	IC	-	ND	IC	IC	-	ND	-	2/7
Ethnicity, white	-	Cor (+)	Cor (+)	IC	-	IC	-	ND	-	ND	-	IC	-	2/7
Marital status, married	-	IC	IC	IC	-	IC	IC	ND	-	-	Det (−)	ND	ND	1/9
Education, higher	IC	Cor (+)	Cor (+)	-	-	IC	-	ND	-	-	-	ND	IC	2/7
Income, higher	IC	NC	Cor (+)	Cor (+)	-	IC	-	ND	-	-	-	IC	IC	2/8
Occupation, blue color	-	IC	IC	-	-	Cor (−)	-	-	Cor (−)	-	-	-	-	2/4
Employment	-	IC	-	-	-	-	Cor (−)	IC	-	IC	IC	ND	IC	1/7
Total work hours	-	-	-	-	-	IC	-	-	Cor (−)	-	-	-	IC	1/3
Overtime work hours	-	-	-	-	-	-	-	-	Cor (−)	-	-	-	-	1/1
Fixed day time work	-	-	-	-	-	-	-	-	-	-	-	-	IC	0/1
Shift work	-	-	-	-	-	-	-	-	-	-	-	-	IC	0/1
Multiple job	-	-	-	-	-	-	-	-	-	-	-	-	IC	0/1
Full time employment	-	-	-	-	-	-	-	-	IC	-	-	-	-	0/1
Retirement	-	-	-	-	-	-	-	-	-	IC	Det (−)	-	-	1/2
Trajectory of employment, downward	-	-	-	-	-	-	-	-	-	-	-	-	IC	0/1
Transition to university	-	-	-	-	-	-	-	-	-	-	Cor (−)	-	-	1/1
Pregnancy	-	-	-	-	-	-	IC	-	-	-	Det (−)	-	-	1/2
Health or fitness status, bad	Cor (−)	Cor (−)	-	-	-	IC	-	Det (−)	-	IC	-	-	-	3/5
Chronic diseases, hypertension, CVD, cancer, and diabetes	-	IC	IC	-	-	-	IC	-	-	IC	-	-	-	0/4
Injury history	-	IC	IC	-	-	-	-	-	-	-	-	-	-	0/2
Childhood illness/disability	-	-	-	-	-	-	IC	-	-	-	-	-	-	0/1
Functional limitation, disability	-	IC	-	-	-	-	-	IC	-	-	-	-	-	0/2
Genetic factor	-	-	Cor (+)	-	-	-	-	-	-	-	-	-	-	1/1
Psychological, cognitive and emotional factors														
Attitude	Cor (+)	-	ND	-	-	IC	-	IC	-	IC	-	IC	IC	1/7
Control over exercise	Cor (+)	-	IC	-	-	Cor (+)	-	-	-	-	-	-	-	2/3
Intention to exercise	Cor (+)	-	Cor (+)	-	-	IC	-	Det (+)	-	IC	-	IC	Det (+)	4/7
Outcome expectations (expect benefit)	Cor (+)	Cor (+)	Cor (+)	-	-	IC	-	IC	-	IC	-	IC	-	3/7
Value of exercise outcomes	-	-	IC	-	-	-	-	-	-	-	-	-	-	0/1
Physical outcome realization	-	-	-	-	-	-	-	Det (+)	-	IC	-	-	-	1/2
Psychological outcome realization	-	-	-	-	-	-	-	Det (+)	-	-	-	-	-	1/1
Health locus of control	IC	-	-	-	-	-	-	IC	-	IC	-	-	-	0/3
Action planning	-	-	-	-	-	-	-	Det (+)	-	-	-	-	-	1/1
Perceived behavioral control	Cor (+)	-	IC	-	-	Cor (+)	-	-	-	IC	-	IC	Det (+)	3/6
Self-efficacy	Cor (+)	Cor (+)	Cor (+)	Cor (+)	-	Cor (+)	-	Det (+)	-	IC	-	IC	Det (+)	7/9
Self-motivation	-	Cor (−)	Cor (+)	-	-	-	-	IC	-	IC	-	-	IC	2/5
Self-schemata for exercise	-	-	Cor (+)	-	-	-	-	-	-	-	-	-	-	1/1
Enjoyment of exercise	IC	IC	Cor (+)	IC	-	-	-	IC	-	-	-	-	-	1/5
Stage of change	IC	-	Cor (+)	-	-	IC	-	Det (+)	-	IC	-	-	-	2/5
Process of psychological change	-	-	Cor (+)	-	-	-	-	-	-	-	-	IC	-	1/2
Knowledge of health and exercise	Cor (+)	Cor (+)	NC	-	-	-	-	IC	-	-	-	IC	IC	2/6
Normative beliefs	-	-	NC	-	-	-	-	-	-	IC	-	-	ND	0/3
Body image	-	-	-	-	-	-	-	-	-	IC	-	-	-	0/1
Psychological health	IC	-	IC	Cor (+)	-	-	-	ND	-	-	-	-	-	1/4
Stress	-	Cor (−)	IC	IC	-	IC	-	Det (−)	-	IC	-	-	ND	2/7
High job strain	-	-	-	-	-	-	-	-	Cor (−)	-	-	-	-	1/1
Barrier to exercise	Cor (−)	-	Cor (−)	-	-	IC	-	IC	-	IC	-	-	IC	2/6
Perceived fitness, good	-	Cor (+)	Cor (+)	Cor (+)	-	-	-	-	-	-	-	-	Det (+)	4/4
Quality of life, good	-	-	-	-	-	-	-	-	-	-	-	-	IC	0/1
Lack of time	-	Cor (−)	Cor (−)	-	-	-	-	-	-	-	-	-	-	2/2
Susceptibility to illness/seriousness of illness	-	-	NC	-	-	-	-	-	-	-	-	-	-	0/1
Fear of symptoms	-	Cor (−)	-	Cor (−)	-	-	-	-	-	-	-	-	-	2/2
Mood disturbance	-	-	Cor (−)	-	-	-	-	-	-	-	-	-	-	1/1
Depression	-	-	-	-	-	-	-	IC	-	IC	-	-	IC	0/3
Fatigue	-	Cor (−)	-	-	-	-	-	-	-	-	-	-	IC	1/2
Anxiety	-	-	-	-	-	-	-	-	-	-	-	-	IC	0/1
John Henryism	-	IC	-	-	-	-	-	-	-	-	-	-	-	0/1
Personality variables	-	-	IC	-	-	-	-	-	-	-	-	-	-	0/1
Extraversion	-	-	-	-	Cor (+)	-	-	-	-	-	-	-	-	1/1
Openness to experience	-	-	-	-	NC	-	-	-	-	-	-	-	-	0/1
Agreeableness	-	-	-	-	NC	-	-	-	-	-	-	-	-	0/1
Neuroticism	-	-	-	-	Cor (−)	-	-	-	-	-	-	IC	-	1/2
Conscientiousness	-	-	-	-	Cor (+)	-	-	-	-	-	-	-	-	1/1
Psychoticism	-	-	-	-	NC	-	-	-	-	-	-	-	-	0/1
Behavioral factors														
Dietary habits	-	IC	Cor (+)	IC	-	IC	-	-	-	-	-	-	-	1/4
Overweight/obesity	-	IC	Cor (−)	-	-	IC	-	ND	-	IC	-	-	-	1/5
Alcohol	-	IC	IC	-	-	-	-	IC	-	-	-	ND	-	0/4
Smoking	-	NC	IC	IC	-	IC	-	IC	-	IC	-	ND	-	0/7
Activity during adulthood	IC	-	Cor (+)	-	-	-	-	Det (+)	-	IC	-	-	-	2/4
Activity during childhood	IC	-	IC	-	-	-	-	-	-	IC	-	-	-	0/3
Past exercise program	-	IC	Cor (+)	-	-	-	-	-	-	-	-	-	-	1/2
Processes of behavioral change	-	-	Cor (+)	-	-	-	-	-	-	-	-	IC	-	1/2
School sports	-	-	IC	-	-	-	-	-	-	-	-	-	-	0/1
Type A behavior pattern	-	IC	IC	-	-	-	-	-	-	-	-	-	-	0/2
Decisional balance sheet	-	-	IC	-	-	-	-	-	-	-	-	-	-	0/1
Screening	-	IC	-	-	-	-	-	-	-	-	-	-	-	0/1
Physical activity intensity	-	-	IC	-	-	-	-	IC	-	-	-	-	-	0/2
Social and cultural factors														
Social support for exercise	-	-	-	Cor (+)	-	-	-	IC	-	IC	-	IC	-	1/4
Social support for exercise from friends/peers	Cor (+)	-	Cor (+)	IC	-	-	-	IC	-	IC	-	-	-	2/5
Social support for exercise from spouse/family	IC	-	Cor (+)	IC	-	-	-	-	-	IC	-	-	-	1/4
Social support from staff/instructor	-	-	-	-	-	-	-	IC	-	IC	-	-	-	0/2
Physician influence	IC	Cor (+)	Cor (+)	IC	-	-	-	IC	-	IC	-	-	-	2/6
Spousal physical activity habits	-	-	-	-	-	-	-	-	-	-	-	-	IC	0/1
Work-family conflict	-	-	-	-	-	-	-	-	-	-	-	-	IC	0/1
Social support	NC	Cor (+)	-	-	-	IC	-	IC	-	-	-	-	-	1/4
Group cohesion	-	-	-	-	-	-	-	IC	-	IC	-	-	IC	0/3
Social isolation	-	-	IC	-	-	-	-	-	-	-	-	IC	-	0/2
Neighborhood deprivation	-	-	-	-	-	-	-	-	-	-	-	-	IC	0/1
Caregiver to ill family member	-	-	-	-	-	-	-	-	-	-	-	-	IC	0/1
Change in family structure: having child	-	Cor (−)	-	-	-	-	-	-	-	-	-	-	Det (−)	2/2

*Abbreviations*: *NC* not a correlate, *ND* not a determinant, *Cor* a correlate, *Det* a determinant, *IC* inconclusive, *DAF* definitely associated factor

^a^Including results of following reviews: “Dishman et al. [39]”, “Dishman et al. [40]”,"Dishman et al. [41]” “Sallis and Owen. 1999”

^b^Number of reviews regarding the factor as definitely associated factor / total number of reviews assessing the factor

Table 3 lists the relationships between environmental factors and physical activity overall. There were 27 environment factors consisting of 4 facility factors, 8 neighborhood factors, 6 safety factors, 3 home environment factors, 3 location of region factors, and 3 climate factors. Among the 27 environmental factors, ten factors were considered as DAFs in more than one of the reviews. For facility factors, accessibility was assessed in more than half of the reviews (10 out of 19) and classified as a positive DAF in 5 reviews. For neighborhood factors, the presence of sidewalks and aesthetics were evaluated in 14 reviews and regarded as positive DAFs in more than three reviews. For safety factors, high crime rates in the region and heavy traffic were only determined as DAFs in less than three reviews although they were summarized in more than half of the reviews. There were no factors which were assessed in more than half of the reviews (10 out of 19) for home environment, location of region, and climate factors.

**Table 3:** Relationships between environmental factors and physical activity

	Eyler (2002) [10]	Trost (2002) ^a^[9]	Humpel (2002) [18]	Plonczynski (2003) [19]	Cunningham (2004) [20]	Owen (2004) [21]	Duncan (2005) [11]	Tucker (2007) [24]	Wendel-vos (2007) [25]	Saelens (2008) [27]	Van Stralen (2009) [28]	Panter (2010) [29]	Koeneman (2011) [33]	McCormack (2011) [30]	Van Cauwenberg (2011) [31]	Van Holle (2012) [35]	Rhode (2015) [36]	Day (2016) [37]	Prince (2016) [38]	No. of DAF/ total No.^b^
Facility																				
Accessibility	-	IC	Cor (+)	-	IC	Cor (+)	Cor (+)	-	Cor (+)	IC	IC	-	IC	IC	IC	IC	ND	Cor (+)	IC	5/15
Convenience of facilities	IC	-	IC	-	IC	Cor (+)	-	-	IC	-	-	-	-	-	-	-	-	-	ND	1/6
Satisfaction with facilities	-	IC	IC	-	-	-	-	-	-	-	-	-	IC	-	-	-	-	-	-	0/3
Cost of programs	-	IC	-	-	-	-	-	-	IC	-	-	-	-	-	-	-	-	-	-	0/2
Neighborhood																				
Presence of sidewalks	IC	IC	Cor (+)	IC	IC	IC	Cor (+)	-	NC	IC	IC	IC	-	IC	IC	-	-	Cor (+)	-	3/14
Aesthetics	IC	IC	Cor (+)	-	Cor (+)	Cor (+)	-	-	IC	IC	IC	IC	-	IC	NC	Cor (+)	ND	IC	-	4/14
Transportation	-	-	-	-	IC	-	-	-	IC	-	-	-	-	IC	-	-	-	-	-	0/3
Sprawl	-	-	IC	-	IC	IC	-	-	IC	-	-	-	-	IC	-	-	-	-	-	0/5
Population density	-	-	-	-	-	-	-	-	-	IC	-	-	-	IC	IC	IC	-	IC	-	0/5
Network connectivity	-	-	-	-	-	-	-	-	-	IC	IC	-	-	IC	IC	IC	-	IC	IC	0/7
Land-use mix	-	-	-	-	-	-	-	-	IC	Cor (+)	IC	-	-	NC	IC	IC	-	Cor (+)	-	2/7
Quality of environment	-	-	-	-	-	-	-	-	-	-	-	-	-	-	-	IC	-	-	-	0/1
Safety																				
High crime rates in the region	IC	IC	IC	-	NC	IC	Cor (+)	-	IC	IC	IC	-	-	-	IC	IC	-	IC	IC	1/13
Heavy traffic	IC	IC	IC	-	NC	IC	Cor (+)	-	IC	IC	IC	IC	-	IC	IC	IC	-	IC	-	1/14
Frequently observe others exercising	IC	IC	-	-	-	-	-	-	IC	-	-	-	-	-	-	-	-	-	-	0/3
Neighborhood safety	Cor (−)	IC	IC	IC	IC	-	-	-	-	-	IC	-	IC	-	IC	IC	-	-	-	1/9
Adequate lighting	IC	IC	IC	-	IC	IC	IC	-	IC	-	IC	-	-	IC	-	-	-	-	-	0/9
Unattended dogs	IC	IC	IC	-	IC	IC	IC	-	IC	-	IC	-	-	-	-	-	-	IC	-	0/9
Home environment																				
Home equipment	-	IC	IC	-	-	IC	-	-	IC	-	IC	-	-	-	-	-	-	-	-	0/5
Home age	-	-	-	IC	IC	IC	-	-	IC	-	-	-	-	-	-	-	-	-	-	0/4
Stair in the home	-	-	-	IC	-	-	-	-	-	-	-	-	-	-	-	-	-	-	-	0/1
Location of region																				
Hilly terrain	IC	IC	IC	-	IC	IC	-	-	IC	-	IC	-	-	-	-	IC	-	-	-	0/8
Coastal location	-	-	-	-	-	IC	-	-	IC	-	-	IC	-	-	-	-	-	-	-	0/3
Urban location	IC	IC	-	IC	-	-	-	-	IC	-	-	-	-	-	IC	IC	-	Cor (−)	IC	1/8
Climate																				
Bad weather	IC	-	IC	IC	-	IC	-	IC	NC	-	-	-	IC	-	-	-	-	IC	-	0/8
Pollution	-	-	-	-	-	-	-	-	IC	-	-	-	-	-	-	-	-	IC	-	0/2
Season, summer	IC	-	-	-	-	-	-	Cor (+)	-	-	-	-	-	-	-	-	-	-	IC	1/3

*Abbreviations*: *NC* not a correlate, *ND* not a determinant, *Cor* a correlate, *Det* a determinant, *IC* inconclusive, *DAF* definitely associated factor

^a^Including results of following reviews: “Dishman et al. [39]”, “Dishman et al. [40]”,"Dishman et al. [41]” “Sallis and Owen. 1999”

^b^Number of reviews regarding the factor as definitely associated factor / total number of reviews assessing the factor

### Correlates of physical activity by the domains of the physical activity

The results by the domains of physical activity are summarized in Additional file 1: Tables S4 ~ S7. For personal factors, the factors for leisure-time physical activity were summarized (Additional file 1: Table S4). Of the 46 personal factors, twenty-two factors were considered as DAFs in one of the reviews. There were no personal factors considered more than twice as a DAF. For environmental factors, factors were summarized in leisure time physical activity, walking/cycling, and transportation, respectively (Additional file 1: Table S5 ~ S7). There were 6 factors regarded as DAFs in more than one of the reviews. Accessibility was considered as a DAF in all three domains. Population density and high crime rate in the region were considered as DAFs only in the leisure-time physical activity domain (Additional file 1: Table S5). Land-use mix and urban location were classified as DAFs in transportation (Additional file 1: Table S6) and walking/cycling (Additional file 1: Table S7). Aesthetics was considered once as a DAF only in the walking/cycling domain (Additional file 1: Table S7). The results for the occupation and household domain could be not summarized for both personal and environmental factors because there was only one or no reviews for those domains.

### Other issues for correlates of physical activity

When summarizing the review of studies conducted in older subjects, no differences were found when compared with the results for all adults. There were 13 personal factors which were classified as DAFs in at least one of two reviews that only focused on factors of older adults (> 65 years) (See the results of Rhode et al. [17] and Plonczynski et al. [19] in Table 2). There were no environmental factors considered as DAFs for older adults.

The results of objectively measured physical activity could be not summarized in this review of reviews because most of the reviews included less than four primary studies using objectively measured physical activity or did not provide information on the measurement of physical activity in the primary studies. In the results of objectively measured environmental factors, the following 5 factors were considered as DAFs in more than one of the reviews from among 17 factors: accessibility, population density, land-use mix, urban location, and high crime rate in the region (Additional file 1: Table S8).

### Corrected Covered Area (CCA)

Additional file 1: Table S9 presents the CCA for each factor. The primary studies had a slight overlap across 13 (CCA: 2.0%) and 18 reviews (CCA: 1.6%) for personal and environmental factors, respectively. In addition, all the CCAs for the factors classified as DAFs in more than 3 reviews were less than 5%.

## Discussion

This review of reviews summarized the results of 25 previous reviews that reported on the potential factors of participation in physical activity all of which showed mostly a moderate methodological quality. Several personal factors including age, health or fitness status, intention to exercise, outcome expectations, perceived behavioral control, self-efficacy, and perceived fitness and several environmental factors including accessibility, presence of sidewalks, and aesthetics were assessed as DAFs in more than three studies.

This study is the first updated review of reviews on factors for physical activity after the study by Bauman in 2012 [13]. Four reviews for personal factors [10, 34, 36, 38] and ten reviews for environmental factors [9, 10, 19, 24, 30, 33, 35–38] were added in the present study after the previous review of reviews [13]. Most factors presented as correlates in the study by Bauman were considered as DAFs in the present study including personal history of physical activity during adulthood which was classified as a DAF in two reviews. Fifty-four factors were additionally summarized which were not evaluated in the review by Bauman. Among them, transition to university, pregnancy, past exercise program, processes of behavioral change, change in family structure, presence of sidewalks, and season were classified as DAFs at least once. A summary of previous reviews by the domains of physical activity and by measures of environmental factors was added.

For personal factors, self-efficacy was consistently evaluated as the clearest correlate in the present study consistent with the previous review of reviews [13]. According to Bandura’s Social Cognitive Theory, self-efficacy functions both directly and indirectly with outcome expectations and other constructs [43] and has a role as a mediating factor of social support in health behavior [44, 45].

For environmental factors, this study summarized the factors by the domains of physical activity, and the results of some factors such as accessibility were consistent overall and by the domains of physical activity. However, it can be concluded that it is too early to summarize the results of the review because there were a limited number of primary studies for each factor.

Although there were a number of factors whose effects on physical activity were assessed, we could not perform a meta-analysis because of the lack of primary studies for each factor, different analytical measures, and the presence of unclearly distinguished factors when compared with each other. For example, some psychological factors had similar definitions such as attitude and outcome expectation. There were many factors classified as a group such as employment-related factors including occupation type, employment status, total work hours, overtime work hour, fixed day time work, shift work, multiple job, and full time employment and social support-related factors including social support for exercise overall and from friends/peers, spouse/family, and staff/instructor and those factors were listed in their originally written form from each review to convey the most accurate meaning of each factor rather than conducting a meta-analysis.

Instead of a meta-analysis, the present study conducted a review of reviews. Although a review of reviews can only show the tendency or direction of an association rather than providing the magnitude or significance level of an association [46], the current evidence on participation in physical activity was comprehensively summarized. When using the review of reviews, there were some challenges. First, the quality of the review of reviews was greatly affected by the quality of the original reviews [47]. In this study, we confirmed that the quality of the original reviews were mostly moderate or higher by assessing the AMSTAR score. Second, if the primary studies were included in several reviews, they may produce bias related to overlapping effects [47]. By calculating the CCA, we showed that the primary studies included in each review were only slightly overlapped and proved that the results from each review were relatively independent.

The present study has limitations. First, a study by Duncan [11] was not included in the calculation of the CCA because it did not provide a list of the included primary studies. However, the effect of not including these primary studies is expected to be slight because there were only 16 primary studies in the study by Duncan. Second, the results of intervention and observational studies could not be separately summarized because the results were not presented separately for each design in most reviews. Further studies should summarize the effects of potential factors on physical activity by the design of the study. Third, policy-related variables were not considered in the present study because policies were rarely considered in previous reviews. Although the effects of policy-related factors were overlapped with the effects of environmental factors such as the presence of sidewalks, the effects of policies on participation in physical activity should be investigated in a future study. Fourth, the interaction effect between different types of factors such as age and presence of sidewalks could not be assessed because the previous reviews were only focused on the individual effect of each factor. Like the interaction effect, the moderating effect of individual factors such as gender and age could not be evaluated also because there were no reviews on this issue. Future research should be conducted to identify the interaction or moderating effect of each factor.

## Conclusion

In conclusion, the present study summarized the associations of potential factors with physical activity which could provide directions for improving participation in physical activity. More studies with a longitudinal design are needed to validate the associations of many factors. If more correlates are established with an accurate method, those factors can be used to form public policies and programs that will encourage the public to participate in physical activity and ultimately improve the public health.

## Additional file

Additional file 1: Table S1. AMSTAR score for each review. **Table S2.** Modified classification of the variables from each review based the evidence from the primary studies. **Table S3.** Definition of each factor. **Table S4.** Relationships between personal factors and leisure-time physical activity. **Table S5.** Relationships between environmental factors and leisure-time physical activity. **Table S6.** Relationships between environmental factors and transportation. **Table S7.** Relationships between environmental factors and walking/cycling. **Table S8.** Relationships between objectively measured environmental factors and physical activity. **Table S9.** CCA for each factor. (DOCX 85 kb)

## Abbreviations

AMSTAR: assessing the methodological quality of systematic reviews
CCA: corrected covered area
Cor: correlate
DAF: definitely associated factor
Det: determinant
IC: inconclusive
MeSH: medical subject heading
NC: not correlate
ND: not determinant
