# Supplementary material for: Correlates associated with participation in physical activity among adults: a systematic review of reviews and update
Source: BMC Public Health. 2017 Apr 24;17:356. doi: 10.1186/s12889-017-4255-2 (PMC5404309; doi:10.1186/s12889-017-4255-2)
Supplement: Additional file 1: Table S1. — AMSTAR score for each review. Table S2. Modified classification of the variables from each review based the evidence from the primary studies. Table S3. Definition of each factor. Table S4. Relationships between personal factors and leisure-time physical activity. Table S5. Relationships between environmental factors and leisure-time physical activity. Table S6. Relationships between environmental factors and transportation. Table S7. Relationships between environmental factors and walking/cycling. Table S8. Relationships between objectively measured environmental factors and physical activity. Table S9. CCA for each factor. (DOCX 85 kb) [file 12889_2017_4255_MOESM1_ESM.docx]

**Additional file 1**

Table S1. AMSTAR^1^ score for each review

|  | 1 | 2 | 3 | 4 | 5 | 6 | 7 | 8 | 9 | 10 | 11 | Total |
| --- | --- | --- | --- | --- | --- | --- | --- | --- | --- | --- | --- | --- |
| Prince (2016) | 1 | 1 | 1 | 1 | 1 | 1 | 1 | 1 | 0 | 0 | 0 | 8 |
| Day (2016) | 1 | 1 | 1 | 0 | 1 | 1 | 0 | 0 | 0 | 0 | 0 | 5 |
| Rhode (2015) | 1 | 1 | 1 | 0 | 1 | 1 | 1 | 0 | 0 | 0 | 0 | 6 |
| Van Holle (2012) | 1 | 1 | 1 | 0 | 1 | 1 | 0 | 0 | 0 | 0 | 0 | 5 |
| Enberg (2012) | 1 | 1 | 1 | 0 | 1 | 1 | 1 | 0 | 0 | 0 | 0 | 6 |
| Koeneman (2011) | 1 | 1 | 1 | 0 | 1 | 1 | 1 | 1 | 0 | 0 | 0 | 7 |
| Kirk (2011) | 1 | 1 | 1 | 0 | 1 | 1 | 1 | 1 | 0 | 1 | 0 | 8 |
| Van Cauwenberg (2011) | 1 | 0 | 1 | 0 | 1 | 1 | 0 | 0 | 0 | 0 | 0 | 4 |
| McCormack (2011) | 1 | 1 | 0 | 0 | 1 | 0 | 0 | 0 | 0 | 0 | 0 | 3 |
| Panter (2010) | 1 | 0 | 1 | 0 | 1 | 0 | 0 | 0 | 0 | 0 | 0 | 3 |
| Van Stralen (2009) | 1 | 0 | 1 | 0 | 1 | 1 | 0 | 0 | 0 | 0 | 0 | 4 |
| Saelens (2008) | 1 | 0 | 1 | 1 | 1 | 1 | 0 | 0 | 0 | 0 | 0 | 5 |
| Allender (2008) | 1 | 0 | 0 | 0 | 1 | 1 | 0 | 0 | 0 | 0 | 0 | 3 |
| Wendel-vos (2007) | 1 | 1 | 1 | 0 | 1 | 1 | 0 | 0 | 0 | 0 | 0 | 5 |
| Tucker (2007) | 1 | 0 | 1 | 0 | 1 | 1 | 0 | 0 | 0 | 0 | 0 | 4 |
| Rhodes (2006) | 1 | 0 | 1 | 0 | 1 | 1 | 0 | 0 | 0 | 0 | 0 | 4 |
| Kaewthummanukul (2006) | 1 | 0 | 1 | 0 | 1 | 1 | 0 | 0 | 0 | 0 | 0 | 4 |
| Duncan (2005) | 1 | 0 | 1 | 0 | 0 | 0 | 0 | 0 | 1 | 0 | 0 | 3 |
| Owen (2004) | 0 | 1 | 1 | 0 | 1 | 1 | 0 | 0 | 0 | 0 | 0 | 4 |
| Cunningham (2004) | 1 | 0 | 1 | 0 | 1 | 1 | 0 | 0 | 0 | 0 | 0 | 4 |
| Plonczynski (2003) | 0 | 0 | 1 | 0 | 1 | 1 | 0 | 0 | 0 | 0 | 0 | 3 |
| Trost (2002) | 0 | 0 | 1 | 0 | 1 | 0 | 0 | 0 | 0 | 0 | 0 | 2 |
| Humpel (2002) | 1 | 0 | 1 | 0 | 1 | 1 | 0 | 0 | 0 | 0 | 0 | 4 |
| Eyler (2002) | 1 | 0 | 1 | 0 | 1 | 0 | 0 | 0 | 0 | 0 | 0 | 3 |
| Rhodes (1999) | 1 | 0 | 1 | 0 | 1 | 0 | 0 | 0 | 0 | 0 | 0 | 3 |
| Median (Q1-Q3) |  |  |  |  |  |  |  |  |  |  |  | 4 (3-5) |
| Mean ± SD |  |  |  |  |  |  |  |  |  |  |  | 4.40 ± 1.55 |

^1^AMSTAR Checklist (Shea et al. BMC Medical Research Methodology 2007 7:10)

1. **Was an 'a priori' design provided?**
2. **Was there duplicate study selection and data extraction?**
3. **Was a comprehensive literature search performed?**
4. **Was the status of publication (i.e. grey literature) used as an inclusion criterion?**
5. **Was a list of studies (included and excluded) provided?**
6. **Were the characteristics of the included studies provided?**
7. **Was the scientific quality of the included studies assessed and documented?**
8. **Was the scientific quality of the included studies used appropriately in formulating conclusions?**
9. **Were the methods used to combine the findings of studies appropriate?**
10. **Was the likelihood of publication bias assessed?**
11. **Was the conflict of interest included?**

Table S2. Modified classification of the variables from each review based the evidence from the primary studies^1^

| Meaning of code | % of primary studies in each variable | No. of primary studies in each variable | Code^2^ |  |  |
| --- | --- | --- | --- | --- | --- |
| No association | 60-100% with non-significant association | ≥4 | NC or ND |  |  |
| Positive association | 60-100% with significantly positive association | ≥4 | Cor (+) or Det (+) |  |  |
| Negative association | 60-100% with significantly negative association | ≥4 | Cor (–) or Det (–) |  |  |
| Inconsistent result | 34-59% of associations or no associations | - | IC |  |  |
|  | 60-100% with any associations | <4 | IC |  |  |

Abbreviations: NC, not a correlate; ND, not a determinant; Cor, a correlate; Det, a determinant; IC, inconclusive

^1^Modified methods of Sallis et al. 2000 [14]

^2^When more than 50 percent of the results were derived from prospective studies, it was coded as determinant: ND, Det (+), or Det (–). Correlate and determinant were considered as a definitely associated factor (DAF)

Table S3. Definition of each factor

| Factor | Definition of factor | Source |
| --- | --- | --- |
| Accessibility | Accessibility of facilities such as cycle path, steep hill, local park, busy street, and shops in walking distance | Humpel |
| Action planning | Implementation intentions which can help initiation by specifying when, where and how to act | Van Stralen |
| Activity during adulthood | Physical activity during adulthood | Trost |
| Activity during childhood | Physical activity during childhood | Trost |
| Adequate lighting | Presence of adequate streetlight in neighborhood | Eyler |
| Aesthetics | Aesthetic nature of the physical environment | Humpel |
| Age | Age of participant | Rhodes (1999) |
| Agreeableness | Tendency to be kind, cooperative, altruistic, trustworthy and generous | Rhodes (2006) |
| Alcohol | High level of alcohol drink | Trost |
| Anxiety | Psychological symptom of anxiety | Prince |
| Attitude | Beliefs that exercise will lead to specific outcomes and evaluation of desirability of such outcomes | Rhodes (1999) |
| Bad weather | Bad or extreme weather such as heavy rain, snowfall, and hail | Tucker |
| Barrier to exercise | Perceived barrier to exercise such as lack of time, laziness and work or social responsibilities. | Rhodes (1999) |
| Body image | Satisfaction with body image | Koeneman |
| Caregiver to ill family member | Caregiving to ill family member | Prince |
| Change in family structure: having child | Having children and family responsibilities | Eyler |
| Childhood illness/disability | Illness and disability during childhood | Allender |
| Chronic diseases, hypertension, CVD, cancer, and diabetes | Health condition with chronic disease | - |
| Coastal location | Residence in coastal location | Owen |
| Conscientiousness | Tendency to be ordered, dutiful, self-disciplined and achievement oriented | Rhodes (2006) |
| Control over exercise | Persons belief of how easy or difficult the performance of the behavior may be’ and is influenced by skills, opportunities and resources | Rhodes (1999) |
| Convenience of facilities | Convenience feeling of facilities for physical activity | Eyler |
| Cost of programs | Cost associated with physical activity | Eyler |
| Decisional balance sheet | Using decisional balance sheet | Trost |
| Depression | Psychological condition with depression | Van Stralen |
| Dietary habits | Healthy dietary habits | Trost |
| Education, higher | Education level of participant | Trost |
| Employment | Status of employment (yes or no) | Eyler |
| Enjoyment of exercise | Degree of enjoyment while doing exercise | Eyler |
| Ethnicity, white | Ethnicity of participant | Trost |
| Extraversion | Tendency to be sociable, assertive, energetic, seek excitement and experience positive affect | Rhodes (2006) |
| Fatigue | Fatigue and its perceived interference with life | Prince |
| Fear of symptoms | Fear of chest pain, symptoms of shortness of breath, myocardial infarction, joint pain, les swelling, and fall | Plonczynski |
| Fixed day time work | Fixed day time work versus those with fixed night time work | Prince |
| Frequently observe others exercising | Neighborhood enable to frequently observe others exercising | Eyler |
| Full time employment | Full time employment versus part-time hours | Kirk |
| Functional limitation, disability | Knee osteoarthritis, pain and accompanying disability in physical function | Van Stralen |
| Gender, men | Gender of participant | Trost |
| Genetic factor | Hereditary | Trost |
| Group cohesion | Extent of connectedness and solidarity among groups in society | Van Stralen |
| Health locus of control | Degrees whether individuals have a belief that they possess some personal control over their health. | Rhodes (1999) |
| Health or fitness status, bad | Physical health such as cardiorespiratory endurance, psychomotor speed, strength, reaction time, functional mobility, and illness | Eyler |
| Heavy traffic | Heavy traffic in the neighborhood | Eyler |
| High crime rates in the region | High crime rates in the region | Eyler |
| High job strain | Level of strain from job | Kirk |
| Hilly Terrain | Residence in hilly location | Owen |
| Home age | Age of home | Wendel-vos |
| Home equipment | Presence of home equipment | Wendel-vos |
| Income, higher | Income level of participant | Trost |
| Injury history | Health condition with injury or past injury history | Eyler |
| Intention to exercise | A weighted appraisal of attitudes towards a behavior and the subjective norms for this behavior | Rhodes (1999) |
| John Henryism | Strategy for coping with prolonged exposure to stresses such as social discrimination by expending high levels of effort which results in accumulating physiological costs. | Eyler |
| Knowledge of health and exercise | Knowledge about health, fitness and exercise behaviors | Rhodes (1999) |
| Lack of time | Perceived lack of time | Eyler |
| Land-use mix | Mixed residential zone with nearby commercial and other uses | Saelens |
| Marital status, married | Marital status of participants | Trost |
| Mood disturbance | Mental disorder such as depression, bipolar disorder, and so on. | Trost |
| Multiple job | Having multiple job | Prince |
| Neighborhood deprivation | Indicator of socioecological status of neighborhood | Prince |
| Neighborhood safety | Including sidewalks, heavy traffic, streetlights, unattended dogs, frequently seeing others exercise, feeling safe, and high crime rate | Eyler |
| Network connectivity | Dense network of street, sidewalk, road, and others | Day |
| Neuroticism | Tendency to be emotionally unstable, anxious, self-conscious and vulnerable | Rhodes (2006) |
| Normative beliefs | Perception of social normative pressures, or relevant others' beliefs that he or she should or should not perform such behavior | Trost |
| Occupation, blue color | Occupation type of participant | Kirk |
| Openness to experience | Tendency to be perceptive, creative, reflective and appreciate fantasy, and aesthetics | Rhodes (2006) |
| Outcome expectations (expect benefit) | Beliefs that exercise will lead to specific outcomes | Rhodes (1999) |
| Overtime work hours | Overtime work hours (e.g. >40 hour per week) | Kirk |
| Overweight/obesity | Obesity | Trost |
| Past exercise program | Physical activity participation both in the past year and before high school | Trost |
| Perceived behavioral control | Component of the theory of planned behaviour, reflects an individual’s perception that they are able to successfully achieve a given task (e.g. increase MVPA) | Prince |
| Perceived fitness, good | Perceived fitness/health | Rhodes (1999) |
| Personality variables | Five factors model defined as neuroticism, extraversion, openness, agreeableness, and conscientiousness | Trost |
| Physical activity intensity | Intensity characteristics of physical activity | Trost |
| Physical outcome realization | Realizations of one’s physical outcome expectations | Van Stralen |
| Physician influence | Social influence from professional physician | Eyler |
| Pollution | Air or noise pollution | Wendel-vos |
| Population density | Population density | Saelens |
| Pregnancy | Changing from pre-pregnancy to pregnancy | Engberg |
| Presence of sidewalks | Presence of sidewalks in neighborhood | Trost |
| Process of psychological change | Psychological stage change in preparation (ready to change), pre-contemplation (not thinking about changing), contemplation (thinking about changing), action (actively engaged in change) and Maintenance (sustaining change over time) | Rhodes (1999) |
| Processes of behavioral change | Behavioral stage change in preparation (ready to change), pre-contemplation (not thinking about changing), contemplation (thinking about changing), action (actively engaged in change) and maintenance (sustaining change over time) | Trost |
| Psychological health | Mental health | Rhodes (1999) |
| Psychological outcome realization | Realizations of one’s physical outcome expectations | Van Stralen |
| Psychoticism | Risk taking, impulsiveness, irresponsibility, manipulativeness, sensation seeking, tough-mindedness and pragmatism | Rhodes (2006) |
| Quality of environment | Quality of neighborhood environment | Van Holle |
| Quality of life, good | Person’s perception of their position in life in the context of that person’s culture and value system and in relation to his/her goals, standards, and concerns | Prince |
| Retirement | Comparison before and after retirement | Engberg |
| Satisfaction with facilities | Satisfaction with facilities | Humpel |
| School sports | Sports activity in school | Trost |
| Screening | More participation in screening | Eyler |
| Season, summer | Four seasons | Tucker |
| Self-efficacy | Individual's beliefs in personal ability to perform an activity | Rhodes (1999) |
| Self-motivation | To do or continue activity without other person's urging or supervision | Eyler |
| Self-schemata for exercise | Self-schemata for exercise | Trost |
| Shift work | Shift work versus regular work | Prince |
| Smoking | Current smoking behavior | Trost |
| Social isolation | Social disconnectedness or isolation | Rhodes (2015) |
| Social support | Any social support from anyone (e.g family, friend, peer, and staff) | Rhodes (2015) |
| Social support for exercise | Social support for physical activity from anyone | Van Stralen |
| Social support for exercise from friends/peers | Social support for physical activity from friends or peers | Van Stralen |
| Social support for exercise from spouse/family | Social support for physical activity from spouse or family | Van Stralen |
| Social support from staff/instructor | Social support for physical activity from staff or instructor | Van Stralen |
| Spousal physical activity habits | Spousal physical activity habits | Prince |
| Sprawl | Low-density residential development; rigid separation of homes, shops, and workplaces | Owen |
| Stage of change | Preparation (ready to change), pre-contemplation (not thinking about changing), contemplation (thinking about changing), action (actively engaged in change) and Maintenance (sustaining change over time) | Rhodes (1999) |
| Stair in the home | Presence of stair in home | Plonczynski |
| Stress | Level of perceived stress | Rhodes (1999) |
| Susceptibility to illness/seriousness of illness | Susceptibility /seriousness of illness | Trost |
| Total work hours | Hours worked or time spent in paid employment | Kirk |
| Trajectory of employment, downward | Those who moved to atypical employees from permanent employees, or unemployed person from permanent or atypical employees | Prince |
| Transition to university | Transition from high school to university | Engberg |
| Transportation | Presence of local transportation system | Wendel-vos |
| Type A behavior pattern | Behavioral pattern of competitiveness, time urgency, and hostility | Trost |
| Unattended dogs | Presence of unattended dogs in neighborhood | Eyler |
| Urban location | Residence in urban location | Plonczynski |
| Value of exercise outcomes | Subjective personal importance of the outcome | Trost |
| Work-family conflict | Conflict for working between family members | Prince |

Table S4. Relationships between personal factors and leisure-time physical activity

|  | Rhodes (1999) | Eyler (2002) | Kirk (2011) | Koenman (2011) | Engberg (2012) | No. of DAF/  total No.^1^ |
| --- | --- | --- | --- | --- | --- | --- |
| No. of included primary studies | 41 | 11 | 62 | 16 | 34 |  |
| *Demographic and biological factors* |  |  |  |  |  |  |
| Age | Cor (-) | IC | - | ND | - | 1/3 |
| Gender, men | Cor (+) | - | IC | IC | - | 1/3 |
| Ethnicity, white | - | IC | - | IC | - | 0/2 |
| Marital status, married | - | IC | - | - | Det (-) | 1/2 |
| Education, higher | IC | IC | - | - | - | 0/2 |
| Income, higher | IC | - | - | - | - | 0/1 |
| Occupation, blue color | - | IC | Cor (-) | - | - | 1/2 |
| Employment | - | IC | - | IC | IC | 0/3 |
| Total work hours | - | - | Cor (-) | - | - | 1/1 |
| Overtime work hours | - | - | Cor (-) | - | - | 1/1 |
| Full time employment | - | - | IC | - | - | 0/1 |
| Retirement | - | - | - | - | Det (-) | 1/1 |
| Transition to university | - | - | - | - | Cor (-) | 1/1 |
| Pregnancy | - | - | - | - | Det (-) | 1/1 |
| Health or fitness status, bad | Cor (-) | - | - | IC | - | 1/2 |
| Functional limitation, disability | - | IC | - | - | - | 0/1 |
|  |  |  |  |  |  |  |
| *Psychological, cognitive, and emotional factors* |  |  |  |  |  |  |
| Attitude | Cor (+) | - | - | IC | - | 1/2 |
| Control over exercise | Cor (+) | - | - | - | - | 1/1 |
| Intention to exercise | Cor (+) | - | - | IC | - | 1/2 |
| Outcome expectations (expect benefit) | Cor (+) | - | - | IC | - | 1/2 |
| Physical outcome realization | - | - | - | IC | - | 0/1 |
| Health locus of control | IC | - | - | IC | - | 0/2 |
| Perceived behavioral control | Cor (+) | - | - | IC | - | 1/2 |
| Self-efficacy | Cor (+) | IC | - | IC | - | 1/3 |
| Self-motivation | - | - | - | IC | - | 0/1 |
| Enjoyment of exercise | IC | - | - | - | - | 0/1 |
| Stage of change | IC | - | - | IC | - | 0/2 |
| Knowledge of health and exercise | Cor (+) | - | - | - | - | 1/1 |
| Normative beliefs | - | - | - | IC | - | 0/1 |
| Body image | - | - | - | IC | - | 0/1 |
| Psychological health | IC | - | - | - | - | 0/1 |
| Stress | - | Cor (-) | - | IC | - | 1/2 |
| High job strain | - | - | Cor (-) | - | - | 1/1 |
| Barrier to exercise | Cor (-) | - | - | IC | - | 1/2 |
| Fear of symptoms | - | Cor (-) | - | - | - | 1/1 |
| Depression | - | - | - | IC | - | 0/1 |
|  |  |  |  |  |  |  |
| *Behavioral factors* |  |  |  |  |  |  |
| Overweight/obesity | - | - | - | IC | - | 0/1 |
| Smoking | - | IC | - | IC | - | 0/2 |
| Activity during adulthood | IC | - | - | IC | - | 0/2 |
| Activity during childhood | IC | - | - | - | - | 0/1 |
|  |  |  |  |  |  |  |
| *Social and cultural factors* |  |  |  |  |  |  |
| Social support for exercise | - | - | - | IC | - | 0/1 |
| Social support for exercise from friends/peers | Cor (+) | - | - | IC | - | 1/2 |
| Social support for exercise from spouse/family | IC | - | - | IC | - | 0/2 |
| Physician influence | IC | - | - | IC | - | 0/2 |
| Social support | NC | Cor (+) | - | - | - | 1/2 |
| Change in family structure: having child | - | IC | - | - | - | 0/1 |

Abbreviations: NC, not a correlate; ND, not a determinant; Cor, a correlate; Det, a determinant; IC, inconclusive; DAF, definitely associated factor

^1^Number of reviews regarding the factor as definitely associated factor / total number of reviews assessing the factor

Table S5. Relationships between environmental factors and leisure-time physical activity

|  | Eyler (2002) | Cunningham (2004) | Koeneman (2011) | Van Cauwenberg (2011)^1^ | Van Holle (2012)^1^ | Day (2016) | No. of DAF/ total No.^1^ |
| --- | --- | --- | --- | --- | --- | --- | --- |
| No. of included primary studies | 1 | 3 | 1 | 14 | 34 | 16 |  |
| *Facility* |  |  |  |  |  |  |  |
| Accessibility | - | IC | IC | Cor (+) | IC | IC | 1/5 |
| Satisfaction with facilities | - |  | IC | - | - | - | 0/1 |
|  |  |  |  |  |  |  |  |
| *Neighborhood* |  |  |  |  |  |  |  |
| Presence of sidewalks | - | IC | - | - | IC | IC | 0/3 |
| Aesthetics | - | IC | - | IC | NC | IC | 0/4 |
| Population density | - | - | - | IC | Cor (-) | - | 1/2 |
| Network connectivity | - | - | - | IC | IC | IC | 0/3 |
| Land-use mix | - | - | - | IC | IC | IC | 0/3 |
| Quality of environment | - | - | - | - | IC | - | 0/1 |
|  |  |  |  |  |  |  |  |
| *Location of region* |  |  |  |  |  |  |  |
| Hilly terrain | - | IC | - | - | - | - | 0/1 |
| Urban location | - | - | - | IC | IC | IC | 0/3 |
|  |  |  |  |  |  |  |  |
| *Safety* |  |  |  |  |  |  |  |
| High crime rates in the region | - | IC | - | Cor (+) | NC | IC | 1/4 |
| Heavy traffic | - | - | - | IC | NC | IC | 0/3 |
| Neighborhood safety | IC | IC | - | - | IC | - | 0/3 |
|  |  |  |  |  |  |  |  |
| *Climate* |  |  |  |  |  |  |  |
| Unattended dogs | - | - | - | - | - | IC | 0/1 |
| Bad weather | - | - | IC | - | - | IC | 0/2 |
| Pollution | - | - | - | - | - | IC | 0/1 |

Abbreviations: NC, not a correlate; ND, not a determinant; Cor, a correlate; Det, a determinant; IC, inconclusive; DAF, definitely associated factor

^1^Number of reviews regarding the factor as definitely associated factor / total number of reviews assessing the factor

Table S6. Relationships between environmental factors and transportation

|  | Panter (2010)^1^ | McCormack (2011)^2^ | Van Cauwenberg (2011)^2^ | Van Holle (2012)^3^ | Day (2016)^4^ | No. of DAF/ total No.^5^ |
| --- | --- | --- | --- | --- | --- | --- |
| No. of included primary studies | 43 | 8 | 6 | 27 | 26 |  |
| *Facility* |  |  |  |  |  |  |
| Accessibility | - | IC | IC | IC | Cor (+) | 1/4 |
|  |  |  |  |  |  |  |
| *Neighborhood* |  |  |  |  |  |  |
| Presence of sidewalks | IC | - | - | IC | IC | 0/3 |
| Aesthetics | IC | IC | IC | NC | IC | 0/5 |
| Population density | - | IC | IC | IC | IC | 0/4 |
| Network connectivity | - | IC | IC | IC | IC | 0/4 |
| Land-use mix | - | IC | IC | IC | Cor (+) | 1/4 |
| Quality of environment | - | - | - | IC | - | 0/1 |
|  |  |  |  |  |  |  |
| *Location of region* |  |  |  |  |  |  |
| Hilly terrain | - | - | - | IC | - | 0/1 |
| Coastal location | IC | - | - | - | - | 0/1 |
| Urban location | - | - | IC | Cor (+) | IC | 1/3 |
|  |  |  |  |  |  |  |
| *Safety* |  |  |  |  |  |  |
| High crime rates in the region | - | - | IC | NC | IC | 0/3 |
| Heavy traffic | IC | IC | IC | IC | IC | 0/5 |
| Neighborhood safety | - | - | - | IC | - | 0/1 |
| Adequate lighting | - | IC | - | - | - | 0/1 |
|  |  |  |  |  |  |  |
| *Climate* |  |  |  |  |  |  |
| Bad weather | - | - | - | - | IC | 0/1 |

Abbreviations: NC, not a correlate; ND, not a determinant; Cor, a correlate; Det, a determinant; IC, inconclusive; DAF, definitely associated factor

^1^Transportation walking/cycling

^2^Transportation walking

^3^General active transportation and transportation walking/cycling

^4^General active transportation

^5^Number of reviews regarding the factor as definitely associated factor / total number of reviews assessing the factor

Table S7. Relationships between environmental factors and walking/cycling

|  | Cunningham (2004) | Owen (2004) | Saelens (2008) | McCormack (2011) | Van Cauwenberg (2011) | Van Holle (2012) | No. of DAF/ total No.^1^ |
| --- | --- | --- | --- | --- | --- | --- | --- |
| No. of included primary studies | 4 | 18 | 29 | 22 | 17 | 33 |  |
| *Facility* |  |  |  |  |  |  |  |
| Accessibility | - | Cor (+) | IC | IC | IC | IC | 1/5 |
| Convenience of facilities | IC | - | - | - | - | - | 0/1 |
| *Home environment* |  |  |  |  |  |  |  |
| Home equipment | - | IC | - | - | - | - | 0/1 |
| Home age | - | IC | - | - | - | - | 0/1 |
|  |  |  |  |  |  |  |  |
| *Neighborhood* |  |  |  |  |  |  |  |
| Presence of sidewalks | - | IC | IC | IC | IC | IC | 0/5 |
| Aesthetics | IC | Cor (+) | IC | IC | IC | NC | 1/6 |
| Transportation | - | - | - | IC | - | - | 0/1 |
| Sprawl | - | IC | - | IC | - | - | 0/2 |
| Population density | - | - | IC | IC | IC | IC | 0/4 |
| Network connectivity | - | - | IC | IC | IC | IC | 0/4 |
| Land-use mix | - | - | Cor (+) | Cor (+) | IC | IC | 2/4 |
| Quality of environment | - | - | - | - | - | IC | 0/1 |
|  |  |  |  |  |  |  |  |
| *Location of region* |  |  |  |  |  |  |  |
| Hilly terrain | - | IC | - | - | - | IC | 0/2 |
| Coastal location | - | IC | - | - | - | - | 0/1 |
| Urban location | - | - | - | - | IC | Cor (+) | 1/2 |
|  |  |  |  |  |  |  |  |
| *Safety* |  |  |  |  |  |  |  |
| High crime rates in the region | - | IC | IC | - | IC | NC | 0/4 |
| Heavy traffic | - | IC | IC | IC | IC | IC | 0/5 |
| Neighborhood safety | IC | - | - | - | IC | NC | 0/3 |
| Adequate lighting | - | IC | - | IC | - | - | 0/2 |

Abbreviations: NC, not a correlate; ND, not a determinant; Cor, a correlate; Det, a determinant; IC, inconclusive; DAF, definitely associated factor

^1^Number of reviews regarding the factor as definitely associated factor / total number of reviews assessing the factor

Table S8. Relationships between objectively measured environmental factors and physical activity

|  | Humpel (2002) | Cunningham (2004) | Owen (2004) | Saelens (2008) | McCormack (2011) | Van Cauwenberg (2011) | Van Holle (2012) | Day (2016) | No. of DAF/ total No.^1^ |
| --- | --- | --- | --- | --- | --- | --- | --- | --- | --- |
| No. of included primary studies | 4 | 4 | 12 | 19 | 31 | 12 | 42 | 30 |  |
| *Facility* |  |  |  |  |  |  |  |  |  |
| Accessibility | IC | IC | IC | IC | IC | NC | IC | Cor (+) | 1/8 |
| *Home environment* |  |  |  |  |  |  |  |  |  |
| Home equipment | - | - | IC | - | - | - | - | - | 0/1 |
|  |  |  |  |  |  |  |  |  |  |
| *Neighborhood* |  |  |  |  |  |  |  |  |  |
| Presence of sidewalks | - | IC | IC | IC | IC | IC | IC | IC | 0/7 |
| Aesthetics | - | IC | - | IC | IC | IC | IC | IC | 0/6 |
| Transportation | - | IC | - | - | IC | - | - | - | 0/2 |
| Sprawl | - | - | IC | - | IC | - | - | - | 0/2 |
| Population density | - | - | - | Cor (+) | IC | IC | IC | IC | 1/5 |
| Network connectivity | - | - | - | IC | IC | IC | IC | IC | 0/5 |
| Land-use mix | - | - | - | IC | NC | IC | IC | Cor (+) | 1/5 |
| Quality of environment | - | - | - | - | - | - | IC | - | 0/1 |
|  |  |  |  |  |  |  |  |  |  |
| *Location of region* |  |  |  |  |  |  |  |  |  |
| Hilly terrain | IC | IC | - | - | - | - | - | - | 0/2 |
| Coastal location | - | - | IC | - | - | - | - | - | 0/1 |
| Urban location | - | - | - | - | - | NC | IC | Cor (-) | 1/3 |
|  |  |  |  |  |  |  |  |  |  |
| *Safety* |  |  |  |  |  |  |  |  |  |
| High crime rates in the region | - | IC | - | IC | - | IC | IC | Cor (-) | 1/5 |
| Heavy traffic | - | IC | IC | NC | IC | IC | IC | IC | 0/7 |
| Neighborhood safety | - | - | - | - | - | IC | IC | - | 0/2 |
| Adequate lighting | - | IC | - | - | IC | - | - | - | 0/2 |
| Unattended dogs | - | IC | - | - | - | - | - | IC | 0/2 |
|  |  |  |  |  |  |  |  |  |  |
| *Climate* |  |  |  |  |  |  |  |  |  |
| Bad weather | - | - | - | - | - | - | - | IC | 0/1 |
| Pollution | - | - | - | - | - | - | - | IC | 0/1 |

Abbreviations: NC, not a correlate; ND, not a determinant; Cor, a correlate; Det, a determinant; IC, inconclusive; DAF, definitely associated factor

^1^Number of reviews regarding the factor as definitely associated factor / total number of reviews assessing the factor

Table S9. CCA for each factor^1^

| Factor | No. of included reviews | No. of included primary studies | CCA (%) |
| --- | --- | --- | --- |
| Personal factors in overall | 13 | 573 | 2.0 |
| Age | 8 | 96 | 1.8 |
| Health or fitness status | 5 | 55 | 4.3 |
| Intention to exercise | 7 | 60 | 1.7 |
| Outcome expectations (expect benefit) | 7 | 36 | 0 |
| Perceived behavioral control | 6 | 42 | 1.2 |
| Self-efficacy | 9 | 116 | 2.0 |
| Perceived fitness | 4 | 36 | 2.8 |
| Environmental factors in overall^2^ | 18 | 413 | 1.6 |
| Accessibility | 15 | 139 | 0.8 |
| Presence of sidewalks | 14 | 65 | 1.7 |
| Aesthetics | 14 | 87 | 1.6 |

^1^CCA calculated for definitely associated factor in more than 3 reviews

^2^Study of Duncan et al. 2005 was excluded because list of included primary studies was not available
